# Attributable fraction of alcohol consumption on cancer using population-based nationwide cancer incidence and mortality data in the Republic of Korea

**DOI:** 10.1186/1471-2407-14-420

**Published:** 2014-06-10

**Authors:** Sohee Park, Hai-Rim Shin, Boram Lee, Aesun Shin, Kyu-Won Jung, Duk-Hee Lee, Sun Ha Jee, Sung-Il Cho, Sue Kyung Park, Mathieu Boniol, Paolo Boffetta, Elisabete Weiderpass

**Affiliations:** 1 Division of Cancer Registration and Surveillance, National Cancer Center, Goyang, South Korea; 2 Department of Biostatistics, Graduate School of Public Health, Yonsei University, Seoul, South Korea; 3 Western Pacific Regional Office, World Health Organization, Manila, Philippines; 4 Department of Preventive Medicine, Seoul National University College of Medicine, Seoul, South Korea; 5 Department of Preventive Medicine, School of Medicine, Kyungpook National University, Daegu, South Korea; 6 Department of Epidemiology and Health Promotion, Institute for Health Promotion, Graduate School of Public Health, Yonsei University, Seoul, South Korea; 7 Graduate School of Public Health and Institute of Health and Environment, Seoul National University, Seoul, South Korea; 8 Department of Biomedical Science, Seoul National University Graduate School, Cancer Research Institute, Seoul National University, Seoul, South Korea; 9 International Prevention Research Institute, Lyon, France; 10 The Tisch Cancer Institute and Institute for Translational Epidemiology, Icahn School of Medicine at Mount Sinai, New York, NY, USA; 11 Cancer Registry of Norway, Oslo, Norway; 12 Department of Community Medicine, Faculty of Health Sciences, University of Tromsø, The Artic University of Norway, Tromsø, Norway; 13 Department of Medical Epidemiology and Biostatistics, Karolinska Institutet, Stockholm, Sweden; 14 Samfundet Folkhälsan, Helsinki, Finland

**Keywords:** Risk factor, Population attributable fraction, Lifestyle, Asia

## Abstract

**Background:**

In the Republic of Korea, cancer is the most common cause of death, and cancer incidence and mortality rates are the highest in East Asia. As alcoholic beverages are carcinogenic to humans, we estimated the burden of cancer related to alcohol consumption in the Korean population.

**Methods:**

The cancer sites studied were those for which there is convincing evidence of a positive association with alcohol consumption: oral cavity, pharynx, esophagus, colon, rectum, liver, larynx and female breast. Sex- and cancer-specific population attributable fractions (PAF) were calculated based on: 1) the prevalence of alcohol drinkers among adults ≥20 years of age in 1989; 2) the average daily alcohol consumption (g/day) among drinkers in 1998; 3) relative risk (RR) estimates for the association between alcohol consumption and site-specific cancer incidence obtained either from a large Korean cohort study or, when more than one Korean study was available for a specific cancer site, meta-analyses were performed and the resulting meta-RRs were used; 4) national cancer incidence and mortality data from 2009.

**Results:**

Among men, 3% (2,866 cases) of incident cancer cases and 2.8% (1,234 deaths) of cancer deaths were attributable to alcohol consumption. Among women, 0.5% (464 cancer cases) of incident cancers and 0.1% (32 deaths) of cancer deaths were attributable to alcohol consumption. In particular, the PAF for alcohol consumption in relation to oral cavity cancer incidence among Korean men was 29.3%, and the PAFs for pharyngeal and laryngeal cancer incidence were 43.3% and 25.8%, respectively. Among Korean women, the PAF for colorectal cancer incidence was the highest (4.2%) and that for breast cancer incidence was only 0.2%. Avoiding alcohol consumption, or reducing it from the median of the highest 4th quartile of consumption (56.0 g/day for men, 28.0 g/day for women) to the median of the lowest quartile (2.80 g/day for men, 0.80 g/day for women), would reduce the burden of alcohol-related cancers in Korea.

**Conclusions:**

A reduction in alcohol consumption would decrease the cancer burden and a significant impact is anticipated specifically for the cancers oral cavity, pharynx, and larynx among men in the Republic of Korea.

## Background

Cancer is the main cause of death in the Republic of Korea, and cancer incidence rates in Korea are the highest in all of East Asia [1,2]. Cancer incidence rates, in particular for cancers of the colon and rectum, breast, thyroid and prostate, have increased significantly during the last 10 years, with an average annual increase of 3.1% for all cancer sites combined [3]. The human, social and economic aspects of the cancer burden are major concerns for the Korean society and its government [4]. Understanding how cancer morbidity and mortality can be prevented, and thereby controlled, would contribute to the country’s public health agenda [5].

According to the Global Status Report on Alcohol and Health, the worldwide consumption of pure alcohol was 6.13 liters/year per person aged 15 years or older in 2005 [6]. There are large geographic differences in consumption patterns, as well as, in most populations, between men and women [6]. Although about half of the world’s population abstains from alcohol consumption, nearly 2 billion adults consume an average of 13 g/day of ethanol (about one drink) [7].

Several studies, mainly among Whites in Europe and North America, have shown that alcohol consumption has a dual effect on mortality, resulting in a U-shaped overall mortality curve, with lifetime non-drinkers and heavy drinkers having the highest overall mortality risk compared to moderate drinkers. This effect reflects the beneficial impact that light to moderate drinking can have on morbidity and mortality due to ischemic heart disease and ischemic stroke, though this beneficial impact disappears with heavy drinking. Nevertheless, alcohol consumption also has deleterious health effects, such as hypertension, cardiac dysrhythmias and hemorrhagic stroke [8]. Therefore, the overall effect of alcohol consumption on the disease burden in a population depends on the distribution of consumption patterns and the background incidence of various alcohol-related diseases. According to the recent evaluation by International Agency for Research on Cancer, there is sufficient evidence that alcohol consumption causes cancers of the oral cavity, pharynx, esophagus, colon, rectum, liver, larynx, and female breast, while evidence for pancreatic cancer is limited. It is known that there is about 10% increased risk of getting breast cancer per alcohol consumption of 10 g/day among women [7,9].

In 2003–2005 the average adult per capita consumption of pure alcohol in Korea was estimated to be 14.8 liters per year, 81% of which was in the form of spirits [6]. The proportion of lifetime non-drinkers in Korea has been decreasing in the past decades among both men (25.4% in 1992; 10.1% in 1998; 12.4% in 2001; 5.3% in 2005) and women (77.4% in 1992; 41.3% in 1998; 38.2% in 2001; 19.2% in 2005) [10]. It was estimated that in 1998 89.9% of Korean men and 58% of Korean women were alcohol drinkers, with a mean consumption of 28.53 g/day among men and 6.38 g/day among women [10]. Further surveys in Korea indicated a decreasing trend in the mean consumption of pure alcohol among men (28.53 g/day in 1998; 26.68 g/day in 2001; 25.33 g/day in 2005), and a slightly increasing trend among women (6.38 g/day in 1998; 5.70 g/day in 2001; 7.92 g/day in 2005) [10].

We conducted a systematic analysis of attributable causes of cancer in Korea, and herein we report estimates of the cancer burden caused by alcohol consumption in the country.

## Methods

### Prevalence of alcohol consumption in Korea

In the present analysis, the prevalence of alcohol consumption in Korea was estimated based on 1) the proportion of alcohol drinkers (the persons who reported non-zero frequency of drinking) among adults aged 20 years or older, and 2) the average alcohol consumption (g/day) among alcohol drinkers. Assuming a latency period of approximately 20 years between alcohol drinking exposure and the cancer occurrence, data from the 1989 Korean National Health Examination Surveys (KNHES) was used to estimate the proportion of alcohol drinkers (Additional file 1: Table S1). Average alcohol consumption (g/day) among alcohol drinkers was calculated based on the type of alcoholic beverage, frequency and amount of usual consumption among individuals who participated in the 1998 Korean National Health and Nutrition Examination Surveys (KNHANES), as this information was not available in the 1989 surveys (Additional file 1: Table S2). Because KNHES and KNHANES data do not contain personal information and are publically available through on-line request (http://knhanes.cdc.go.kr/knhanes/), we did not have to address ethical concerns.

### Identification of Korean studies on alcohol consumption and cancer

Studies reporting relative risks (RRs) of alcohol consumption and cancer in Korean populations, and published through August 1, 2012 were identified using the databases PubMed (http://www.ncbi.nlm.nih.gov/pubmed/) and KoreaMed (http://www.koreamed.org/SearchBasic.php). The search keywords were “Korea”, “alcohol”, “risk”, and “cancer.” Language was limited to English or Korean. At least two independent investigators performed literature search and reviewed articles. We also reviewed references cited from retrieved articles to identify additional studies for inclusion. When there were multiple reports of a single study, the publication with the longest follow-up period or the largest number of cases was selected. When necessary, we also obtained additional data through personal communication with the authors of the studies.

Twenty six studies were initially identified [11-35], 14 of which were subsequently excluded because only the RR for drinkers vs. non-drinkers (defined differently in each study) was available, and the dose–response relationship could not be ascertained. One study was excluded because the number of cases for each exposure category was not presented [13] and another study was excluded due to an overlapping study populations [17]. Additional RR results from updated data with a longer follow-up period (until December 2006), and analyses adjusting for further confounding variables such as age and smoking, were obtained through personal communication [17,18]. Therefore, ten studies, including a large-scale population-based prospective study, were used in the final calculation of RRs for alcohol consumption as they provided the necessary information to assess the dose–response relationship between alcohol consumption and cancer [11,14,15,19,22,25,28,29,31,33].

### Relative risk estimates of cancer according to alcohol consumption in Korea

RRs of alcohol-related cancers per 1 g/day increase in alcohol consumption were estimated by a dose–response analysis of the ten selected studies described above. With different categories of alcohol intake in different studies, we fit a log-linear regression by taking the log of relative risk (or odds ratio) as Y values and taking the midpoint of alcohol consumption for each category as X values to estimate the slope β (log(RR)) for dose–response relationship. From each study, the log(RR) per one gram increase of alcohol consumption on a continuous scale was estimated, then they were used for meta-analysis to estimate the summary log(RR) per one gram increase of alcohol consumption. Because the relationship between the alcohol consumption and relative risk is better captured in a log-linear relationship instead of a linear relationship, the dose–response relationship between alcohol consumption and RR was estimated through log(RR).

A meta-analysis was performed to estimate the pooled RRs for average alcohol consumption based on relevant studies. Separate RRs were estimated for cancer incidence and mortality where possible and when the RR for cancer mortality was not available, the RR for cancer incidence for each cancer site was used for cancer mortality. In cases of heterogeneity across studies, as examined by I^2^ (I^2^ ≥ 80) and Q statistics (p < 0.05), the risk estimates from a random-effects model were used [36]. Publication bias was checked by funnel plot and Begg’s test. The “Metan” command in Stata (version 11.0; StataCorp, College Station, Texas, USA) and Comprehensive Meta-Analysis version 2 (Biostat, Englewood, New Jersey, USA) were used to perform meta-analyses.

### Alcohol-associated cancer sites and data sources for cancer cases and deaths

Cancer sites for which convincing evidence of a positive association with alcohol consumption exists [7,37], and for which RR estimates in Korea were available, were considered in this report: oral cavity, pharynx, esophagus, colon, rectum, liver, larynx and female breast. The number of incident cancer cases in 2009 at sites included in this report was obtained from the Korean Central Cancer Registry, a population-based nationwide cancer registry which is described in more detail elsewhere [3]. The number of cancer deaths in 2009 was obtained using death certificate data from the Korean National Statistics Office [1]. Because we used the aggregated data that do not contain personal information and that are publically available through website (http://www.cancer.go.kr for cancer incidence statistics; and http://www.kosis.kr for cancer mortality statistics), we did not have to address ethical concerns.

### Estimation of population attributable fraction

The sex- and cancer site-specific population attributable fraction (PAF) for alcohol consumption for the year 2009 in Korea was calculated by the following Levin’s formula [38], but applied for continuous exposure [39]:

$$\mathrm{PAF}=\frac{P\left(\mathrm{RR}-1\right)}{P\left(\mathrm{RR}-1\right)+1}=\frac{P\left(e^{\beta *\mathrm{dose}}-1\right)}{P\left(e^{\beta *\mathrm{dose}}-1\right)+1},$$

where β = log(RR) , and the RR corresponds to that associated with a specific cancer site for a 1-unit increase in alcohol consumption, P is the proportion of alcohol drinkers and *dose* is the average alcohol consumption (in g/day as a continuous variable) among drinkers in the total population.

**Figure 1:**
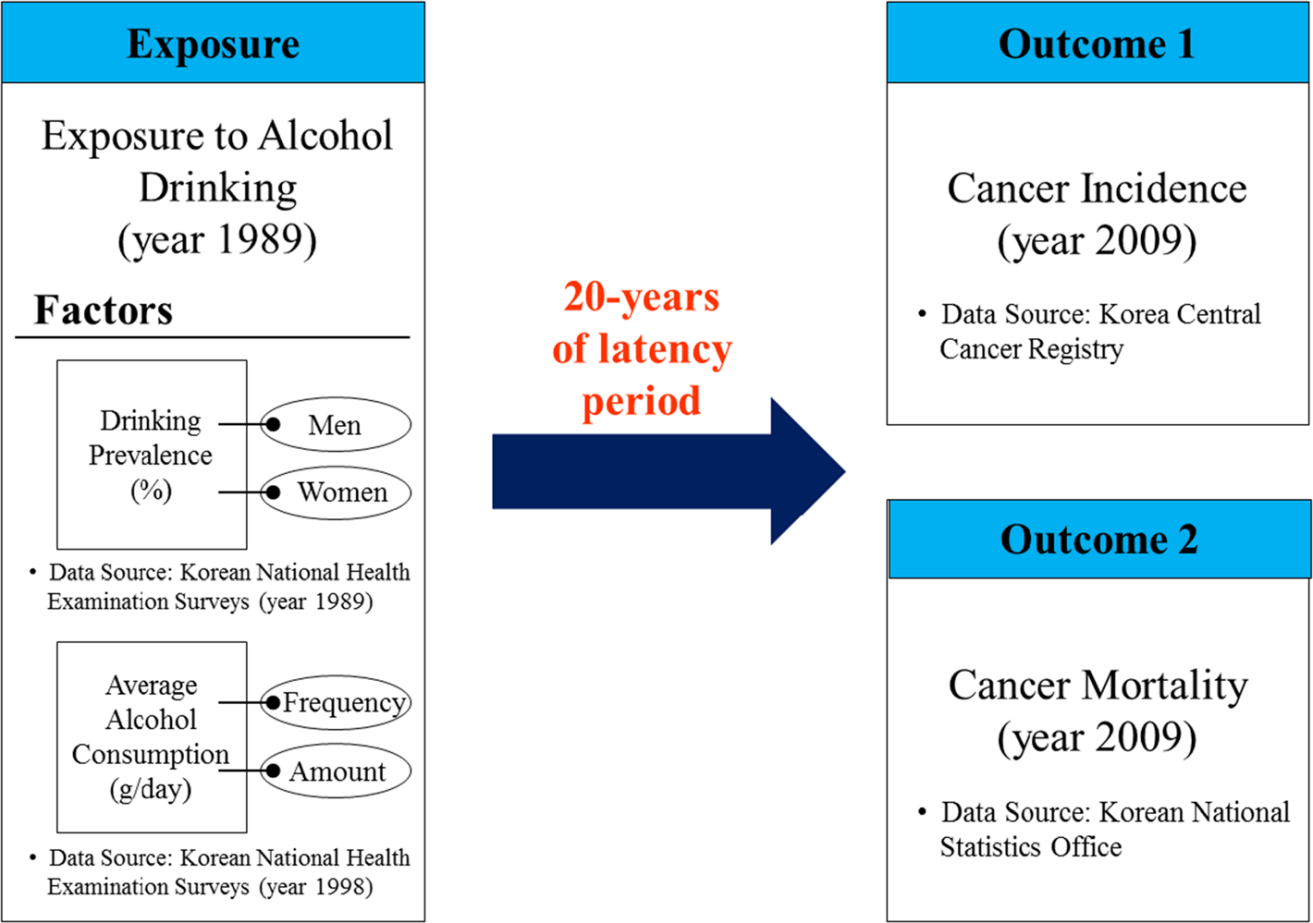
Description of research flow for the PAF calculation and relevant data sources.

Where possible, we estimated the PAFs for cancer incidence and mortality separately. However, we used the same RR for cancer incidence and mortality, that is, we assumed that alcohol drinking has no effect on cancer survival. The Description of research flow for the PAF calculation and relevant data sources are presented in Figure 1. Using the conventional Delta method [40,41], we computed 95% confidence intervals (CI) for the PAF estimates, which turned out to be very narrow for all estimates. Hence, we did not include these in our results, and instead presented the sensitivity analysis as described below.

### Sensitivity analysis and analysis of changes in the population attributable fraction by different alcohol consumption prevalence scenarios

Sensitivity analyses were performed on the PAFs for alcohol consumption using the lower and upper bounds of the 95% CIs of RR estimates. We also investigated the changes in PAF under two hypotheses: 1) that all individuals had high alcohol consumption (4th quartile) and 2) that all individuals had low alcohol consumption (1st quartile) within each sex.

## Results

### Relative risks estimates of cancer according to alcohol consumption in Korea

The estimated RRs of cancer for the studies included in the present report are shown in Table 1, and a complete summary of these studies is shown in Additional file 1: Table S3. For all cancer sites included in this report, alcohol consumption was considered among individuals aged 20 years or older. However, for colon cancer there were additional studies carried out in 1993–1998 concerning alcohol consumption at 65 years of age or older, and for liver cancer an additional case–control study carried out in 1990–1993 reported on alcohol consumption at age 39 years or older. The pooled RRs corresponding to average alcohol consumption for cancer of the oral cavity, pharynx, and larynx were high, ranging from 1.45 to 1.98 in men. Among women, the pooled RR for average alcohol consumption was highest, though not significant, in colorectal cancer (pooled RR = 1.19), followed by pharyngeal cancer (RR = 1.17) and oral cavity cancer (pooled RR = 1.10) (Table 1, Additional file 1: Table S3).

**Table 1:** Estimated relative risks (RR) and 95% confidence intervals (CI) of cancer by sex in Korea

**Cancer site (ICD-10 code)**	**Men**		**Women**		**Source of Pooled RR or OR**
	**Log (Risk per g/day)**	**RR for average**^**d **^**consumption**	**Log (Risk per g/day)**	**RR for average**^**d **^**consumption**	
**Incidence**					
Oral cavity (C00-09)	0.015	1.53 (0.77-2.96)	0.015^a^	1.10 (0.94-1.27)	[17]
					[12]
Pharynx (C10-C14)^b^	0.024	1.98 (1.62-2.42)	0.024^a^	1.17 (1.11-1.22)	[12]
Esophagus (C15)	0.004	1.12 (1.00-1.26)	0.004^a^	1.03 (1.00-1.05)	[17]
					[22]
Colon (C18)	0.004	1.12 (0.80-1.53)	0.027	1.19 (0.88-1.60)	[17]
					[25]
					[19]
Rectum (C20)^b^	0.004	1.12 (0.80-1.53)	0.027	1.19 (0.88-1.60)	[17]
					[25]
					[19]
Liver (C22)	0.002	1.06 (1.03-1.09)	0.002^a^	1.01 (1.01-1.02)	[17]
					[28]
Larynx (C32)	0.013	1.45 (0.82-2.56)	0.013^a^	1.09 (0.96-1.23)	[17]
					[12]
Breast in women (C50)	-	-	0.001	1.01 (0.99-1.02)	[14]
					[33]
**Mortality**					
Oral cavity (C00-09)	0.012	1.41 (1.22-1.67)	0.012^a^	1.08 (1.05-1.12)	[18]
Pharynx (C10-C14)	0.012	1.41 (1.22-1.67)	0.012^a^	1.08 (1.05-1.12)	[12]
Esophagus (C15)	0.010	1.33 (1.12-1.62)	0.010^a^	1.07 (1.03-1.11)	[18]
					[22]
					[29]
					[34]
Colon (C18)	0.002	1.06 (0.94-1.19)	0.002^a^	1.01 (0.99-1.04)	[18]
					[29]
Rectum (C20)^b^	0.002	1.06 (0.94-1.19)	0.002^a^	1.01 (0.99-1.04)	[18]
					[29]
Liver (C22)	0.003	1.09 (1.06-1.15)	0.003^a^	1.02 (1.01-1.03)	[18]
					[29]
Larynx (C32)	0.009	1.29 (1.09-1.49)	0.009^a^	1.06 (1.02-1.09)	[18]
Breast in women (C50)	-	-	0.001^c^	1.01 (0.99-1.02)	[14]
					[33]

Notes: Reference category = non-drinkers (never drinkers + former drinkers).

^a^Using log(RR) in men. ^b^Using log(RR) of colon. ^c^Using log(RR) for cancer incidence in women. ^d^Average alcohol consumption: 28.53 g/day in men, 6.38 g/day in women (1998 National Health and Nutrition Examination Survey) [42,43].

*Abbreviations*: *ICD* International Classification of Diseases.

### Population attributable fraction

**Figure 2:**
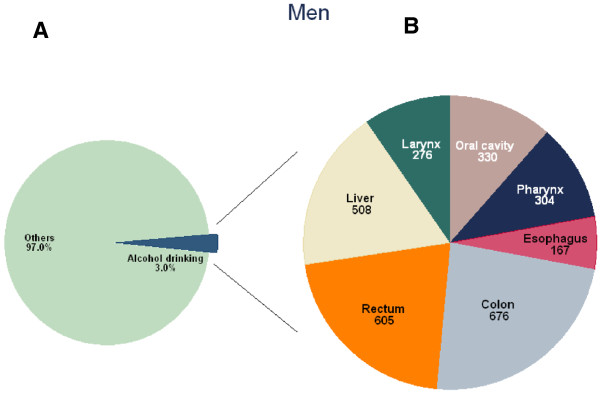
Number of cancer incident cases attributable to alcohol consumption in Korean men, 2009*. * **A)** Proportion of cancer incident cases attributable to alcohol consumption; **B)** Number of cancer incident cases attributable to alcohol consumption by cancer sites.

**Figure 3:**
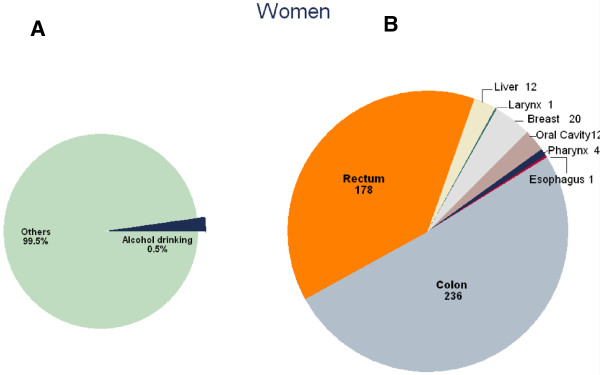
Number of cancer incident cases attributable to alcohol consumption in Korean women, 2009*. * **A)** Proportion of cancer incident cases attributable to alcohol consumption; **B)** Number of cancer incident cases attributable to alcohol consumption by cancer sites

The PAF for alcohol consumption, the number of incident cancer cases and cancer deaths attributable to alcohol consumption overall and by sex are shown in Table 2 by cancer site. The PAF was higher in men (3.0%; 2,866 incident cancer cases, 2.8%, 1,234 cancer deaths) (Figure 2A) than in women (0.5%; 464 incident cancer cases and of 0.1%, 32 cancer deaths) (Figure 3A).

**Table 2:** **PAF and number of cancer cases and cancer deaths**^a ^**attributable to alcohol consumption, Korea, 2009**

**Cancer site (ICD-10 code)**	**Men**						**Women**					
	**PAF**	**No. of cases**	**Alcohol -related cases**	**PAF**	**No. of deaths**	**Alcohol -related deaths**	**PAF**	**No. of cases**	**Alcohol -related cases**	**PAF**	**No. of deaths**	**Alcohol -related deaths**
Oral cavity (C00-C09)	29.3	1,128	330	24.0	397	95	2.3	512	12	1.8	153	3
Pharynx (C10-C14)^b^	43.3	703	304	24.0	368	88	3.7	114	4	1.8	55	1
Esophagus (C15)	8.6	1,947	167	20.4	1,297	264	0.6	175	1	1.5	109	2
Colon (C18)	8.6	7,886	676	4.4	2,206	96	4.2	4,643	236	0.3	1,978	6
Rectum (C20)^c^	8.6	7,063	605	4.4	1,722	75	4.2	3,458	178	0.3	1,142	3
Liver (C22)	4.4	11,663	508	6.5	8,422	545	0.3	3,539	12	0.5	2,814	13
Larynx (C32)	25.8	1,071	276	18.5	382	71	2.0	56	1	1.4	1,878	1
Female breast (C50)	-	-	-		-	-	0.2	11,536	20	0.2	395	3
Total		96,826	2,866		43,658	1,234		91,068	464		25,773	32
% of all cancers			3.0			2.8			0.5			0.1

Notes: ^a^Cases and deaths are from adults (aged 20 years and older). ^b^Using relative risk of oral cavity. ^c^Using relative risk of colon.

*Abbreviations*: *ICD* International Classification of Diseases, *PAF* population attributable fraction.

**Figure 4:**
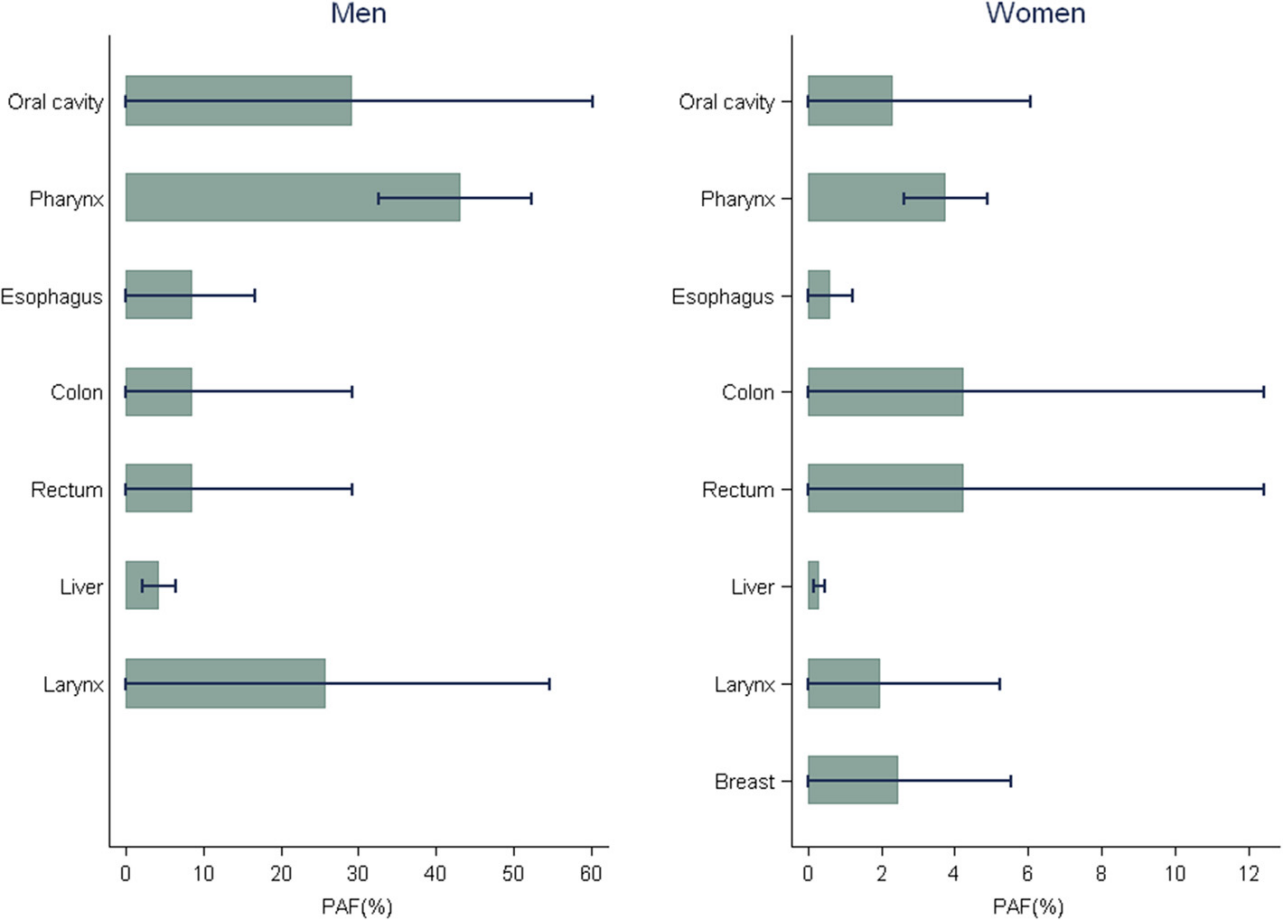
Sensitivity analysis of the PAF for alcohol consumption*. *Lower and upper bounds of 95% CIs for RR estimates used. PAF: population attributable fraction, CI: confidence interval, RR: relative risk.

Among men, the PAF of cancer incidence for alcohol consumption was particularly high in relation to pharyngeal (43.3%), oral cavity (29.3%), laryngeal (25.8%), esophageal (8.6%) and colorectal (8.6%) cancers, and relatively lower for liver cancer (4.4%) (Table 2). However, given the differences in the underlying incidence between cancer sites, the total number of avoidable incident cancer cases in men was largest for colon (676), followed by rectal (605), liver (508), oral cavity (330), pharyngeal (304) and laryngeal (276) cancer (Figure 2B). The PAF of cancer mortality for alcohol consumption was also the highest in oral cavity (24%) and pharyngeal (24%) cancer, followed by esophageal (20.4%), laryngeal (18.5%), liver (6.5%) and colorectal (4.4%) cancer. The total number of avoidable deaths in men was 545 for liver, 264 for esophageal, 71 for laryngeal, 96 for colon, 75 for rectal, 95 for oral cavity and 88 for pharyngeal cancers (Table 2).Among women, the cancer site with the highest number of cases that could have been prevented by avoidance of alcohol consumption was colorectal cancer (414 cases), followed by breast (20 cases), oral cavity (12 cases), liver (12 cases), oral cavity (12 cases) and pharyngeal (4 cases) cancer (Figure 3B). Figure 4 shows the results of the sensitivity analysis of the PAFs for alcohol consumption using the lower and upper bounds of the 95% CIs of RR estimates. The upper bound of the CI for female pharyngeal cancer was 5.0%, that for breast cancer was close to 5.5% and that for female colorectal cancer was close to 12.0% (Figure 4). There has been wide variation due to uncertainty of the RR estimates.

### Changes in population attributable fraction by different alcohol consumption prevalence scenarios

Table 3 shows the PAF calculations using different hypothetical scenarios of alcohol consumption, namely the overall median (men: 28.53 g/day, women: 6.38 g/day), as well as the median of the highest (4th) quartile of consumption (men: 56.0 g/day, women: 28.0 g/day) and median of the lowest (1st) quartile (men: 2.80 g/day, women: 0.80 g/day) [10]. This analysis indicated that if all alcohol drinkers were to consume the same median amount of alcohol as people in the lowest quartile of alcohol consumption, as opposed to the median amount consumed by those in the highest quartile, a substantial proportion of incident cancer cases, and consequently cancer deaths, would be avoided. This potential risk reduction would apply to both sexes. For example, the PAF for alcohol consumption in men would decrease from 50.5% to 3.2% for oral cavity cancer, from 68.7% to 5.1% for pharyngeal cancer, and from 45.4% to 2.8% for laryngeal cancer. Among women, the PAF for alcohol consumption would be most substantially reduced for colon and rectal cancer (from 21% to 0.5%), but the reduction would also be noticeable for pharyngeal cancer (from 18.4% to 0.5% for each). Furthermore, if Korean male drinkers reduced their alcohol consumption, such as beer or *soju* (Korean rice wine), by one glass (approximately 12 g) per day, the total cancer burden attributable to alcohol consumption would be reduced approximately by 1.7%, which implies that we could save about 1,617 cancer patients.Sensitivity analysis showed that the PAF estimates were more sensitive due to higher uncertainty in RR estimates in oral cavity, colorectal and laryngeal cancers. However, for pharyngeal and liver cancers, the PAF estimates were less sensitive (Figure 4).

**Table 3:** PAF for alcohol consumption by different consumption scenarios

**Cancer site**	**Men**				**Women**			
	**PAF(%)**^**a**^	**PAF (Q4)**^**b**^	**PAF (Q1)**^**c**^	**PAF (Q4-Q1)**	**PAF(%)**^**a**^	**PAF (Q4)**^**b**^	**PAF (Q1)**^**c**^	**PAF (Q4-Q1)**
Oral cavity	29.3	50.5	3.2	47.3	2.3	10.9	0.3	10.6
Pharynx	43.3	68.7	5.1	63.6	3.7	18.4	0.5	17.9
Esophagus	8.6	16.3	0.9	15.4	0.6	2.7	0.1	2.6
Colon	8.6	16.3	0.9	15.4	4.2	21.0	0.5	20.5
Rectum^d^	8.6	16.3	0.9	15.4	4.2	21.0	0.5	20.5
Liver	4.4	8.4	0.4	8.0	0.3	1.3	0.0	1.3
Larynx	25.8	45.4	2.8	42.6	2.0	9.4	0.3	9.1
Breast	-				0.2	0.7	0.0	0.6

^a^Men: 28.53 g/day, women: 6.38 g/day (’98 Korea National Health and Nutrition Examination Survey) [42,43].

^b^Median alcohol consumption in the highest (4th) quartile was 56.0 g/day for men and 28.0 g/day for women.

^c^Median alcohol consumption in the lowest (1th) quartile was 2.80 g/day for men and 0.80 g/day for women.

^d^Using RR of colon.

*Abbreviations*: *PAF* Population attributable fraction, *Q* Quartile.

## Discussion

To our knowledge, this is the first study to estimate the PAF for alcohol consumption in relation to cancer risk in Korea, using both alcohol consumption estimates and cancer-related RRs from Korean studies. Our estimates therefore take into account aspects such as the specific carcinogenic effect of alcohol consumption in Korea, and the genetic susceptibility of this population.

As expected, we found that the PAF for alcohol consumption in Korean men was higher than that in Korean women. Among men, 3% of incident cancer cases and 2.8% of cancer deaths were attributable to alcohol consumption. Among the cancer sites included in this report, the PAF for alcohol consumption in men was the largest in relation to pharyngeal cancer (43.3%) and oral cavity (29.3%). However, colorectal and liver cancers had the highest number of avoidable incident cancer cases and deaths due to their high incidence in Korea. Among women only 0.5% of incident cancer cases and 0.1% of cancer deaths were attributable to alcohol consumption; the main cancer types that could have been prevented were colorectal cancer (4.2%), followed by pharyngeal cancer (3.7%).

If changes in patterns of alcohol consumption were to occur in Korea, with people in the highest quartile of alcohol consumption diminishing their consumption to the level of those in the lowest quartile, a substantial decrease in the PAF would occur, most notably among men, decreasing by 64% the number of pharyngeal cancer cases, decreasing by 47% of oral cavity cancer cases, and decreasing by 43% the number of laryngeal cancer cases. Among women, the reductions in risk would be more modest, but still substantial, in particular for colorectal and pharyngeal cancers. For colorectal cancer, reductions in incidence could be up to 20% if we consider the upper bound of the 95% CIs of the PAF estimates.

In our study, the PAF estimates for alcohol consumption in relation to cancer incidence and mortality in Korea were generally lower than corresponding estimates elsewhere. Boffetta et al. [44] estimated a PAF for alcohol consumption in relation to cancer incidence worldwide of 3.6% (5.2% in men, 1.7% in women) and 3.5% for cancer mortality (5.1% in men, 1.3% in women). Other worldwide estimates for cancer mortality indicated a PAF for alcohol consumption of 5% for all cancers combined [6,45]. A few studies in specific populations using a methodology similar to ours have been published recently. A study in China considered cancers of the oral cavity, pharynx, esophagus, colon-rectum, liver and larynx, which have a firmly established association with alcohol consumption. Their PAF estimate for alcohol consumption in relation to overall cancer incidence was 3.63% (5.92% for men and 0.31% for women), and 4.40% (6.69% in men, 0.42% in women) for overall cancer mortality [46]. In France the PAF for cancer mortality was estimated to be 6.9% (9.4% in men and 3.0% in women) [47]. A study conducted in the Russian Federation using a different methodology [48] indicated a much higher PAF for alcohol consumption in relation to several cancer sites due to the fact that mean alcohol consumption in the country is 26.71 liters of pure alcohol per person per year.

As in the studies performed in China [46] and France [47], we only considered cancers for which a firmly established association with alcohol consumption exists [7]. In particular, pancreatic cancer was not considered in our PAF estimates, as the association with alcohol consumption is still not considered to be firmly established, despite several studies suggesting an increased risk of this cancer among alcohol drinkers [7,49]. Therefore our estimate may be somewhat lower than the real PAF for alcohol consumption in relation to cancer incidence in Korea.

Differences between our PAF estimates for Korea and those found elsewhere may be caused both by differences in the methodologies used (for example the RR used for incidence and mortality calculations for each cancer site varied substantially between the studies included in this report), and by patterns of alcohol consumption, namely the proportion of drinkers and non-drinkers, and, among drinkers, the mean amounts consumed [6,10,44-46,50]. Our PAF estimates were based on the proportion of alcohol drinkers over 20 years of age in Korea in 1989 and the estimated daily alcohol consumption in 1998 (mean 28.53 g/day for men, and 6.38 g/day for women) [10], as well as on the RR of cancer incidence and mortality according to alcohol consumption in Korea, which is different than elsewhere (e.g., China). The World Health Organization Global Status Report on Alcohol and Health [6] provides estimates of alcohol consumption in different countries, as well as a pattern of drinking score, which is an estimation of alcohol-related health risks. When comparing alcohol consumption patterns in China (5.6 liters of pure alcohol per person per year; 28% of lifetime non-drinkers; 57% of consumption from spirits; alcohol use disorders 6.90% in men) and Korea (14.8 liters of pure alcohol per person per year; 12.8% of lifetime non-drinkers [5.1% of men and 20.4% of women]; 12.0% of men and 38.9% of women were non-drinkers the year before the survey between 2001 and 2005 [6]; 81% of consumption from spirits; alcohol use disorders 13.10% in men), one could expect a higher PAF in Korea than China. However, this was not observed given the different cancer incidence and mortality patterns, as well as the different RR for alcohol consumption and cancer utilized and the cancer sites included in PAF estimates.

The lower PAF for alcohol consumption in Korea compared to other countries, particularly Western countries, may be partly due to the fact that a large proportion of the Korean population are slow metabolizers of acethaldehyde, a genotoxic substance formed endogenously from alcoholic beverages. Aldehyde dehydrogenases (ALDH) is the main enzyme responsible for detoxifying aldehyde, and maintaining low levels of acetaldehyde during ethanol oxidation [51]. Inactive ALDH2 enzyme is caused by a mutant of ALDH2, known as the ALDH2*2 variant allele. Both individuals homozygous and heterozygous for ALDH2*2 are ALDH2-deficient, but homozygous individuals have higher acetaldehyde levels after they drink alcohol [52]. Accumulation of acetaldehyde after drinking alcohol results in a flushing reaction, which is common in Koreans, Japanese and Chinese, but not Whites [53]. About 30% of East-Asian populations have the ALDH2*2 variant allele, and therefore usually avoid drinking alcohol, or drink lower quantities than other population groups. Cancer risk, particularly esophageal cancer risk, but possibly that of other cancers as well, is increased in people who are slow metabolizers of acethaldehyde. It is possible that some self-selection takes place, where individuals with a flush reaction (who are thus more susceptible to cancer) drink less alcohol [9].

Another possible explanation for the lower PAF for alcohol consumption in Korea compared to Western countries may be that the number of cancers attributable to other factors such as smoking and infection is high. Hence the relative proportion of cancers attributable to alcohol consumption may appear smaller than that in other countries when PAF is computed by dividing the alcohol-related cancers by the total number of cancers, though the attributable fraction may not be as low compared to other countries. Furthermore, it is possible that the PAF for alcohol consumption in Korea has been underestimated due to the use of self-reported alcohol consumption.

A few studies have been published on alcohol consumption and breast cancer risk among Asian women, but their results are inconsistent [9]. Based on studies mainly among Whites, it has been estimated that the RR of female breast cancer increases with increasing alcohol consumption by about 7% per 10 g/day; the estimates were somewhat lower (5%) in the pooling of prospective cohort studies compared to estimates from population-based (7.3%) or hospital-based (7.4%) case–control studies [54]. In a new large study from the United Kingdom including mainly Whites, the risk estimates per 10 g/day of pure alcohol were 12% (95% CI 9-14%) [55].

Alcohol consumption could increase breast cancer risk by altering endogenous hormone levels. Studies among Whites indicate that consumption of over 20 g/day of pure alcohol substantially increases levels of estradiol, free estradiol, estrone, androstenedione, testosterone and free testosterone, while decreasing levels of SHBG [56]. The association between alcohol consumption and endogenous hormones in Asian populations has not been described in as much detail as in Whites; it is biologically plausible that the effect would be different given similar doses of alcohol because of genetic variations and other factors that may influence metabolism (proportion of water to lean body mass, for example) [9].

Our study has both methodological strengths and weaknesses. We based our PAF estimates for alcohol consumption on the proportion of alcohol drinkers 20 years of age or older in 1989, and the average alcohol consumption among drinkers (g/day) in 1998 obtained from well designed and well conducted population-based surveys in Korea [10]. We chose to combine information from these two time periods, as information on average alcohol consumption was not available in the 1989 survey. We assumed that the combination of data on alcohol consumption from 1989 and 1998 was representative of the period of carcinogenesis related to alcohol consumption, i.e., an approximately 20-year latency period between mean exposure to alcohol and cancer incidence and mortality in 2009. However, we cannot rule out the possibility of under-reporting of alcohol consumption, in particular among heavy drinkers, which is common in the assessment of alcohol consumption in questionnaire-based surveys. While there is possibility of underestimating the PAF for alcohol consumption assessed by self-reporting, we believe the underestimation through self-administered questionnaire would not be large, because the Korean society has high tolerance level for heavy alcohol drinking and drinkers are relatively well accepted by Korean social norm [57].

There were a few published epidemiologic studies on alcohol consumption and cancer in Korea which contained risk estimates we could in our study: Studies from one large-scale multi-cancer-site population-based cohort study [17,18,22], two medium-sized cohort studies [25,29], and several hospital-based case–control studies: one on liver cancer with 203 cases [28]; two studies on breast cancer, one with 108 cases [14], and another with 4,508 cases and which had updated data analysis performed by the author [33]; and one study on colorectal cancer with 596 cases [19]. Among them, the one cohort study is a population-based prospective cohort study with over 1 million subjects that can be considered representative of the entire adult Korean population [17,18]. Furthermore, the calculation of RRs of alcohol consumption in the aforementioned study [17,18] was based on a statistical model that adjusted for age and tobacco smoking. Therefore the confounding effect that smoking might have on alcohol consumption was resolved in our RR estimation.

Misclassification of alcohol consumption in both the cohort and case–control studies could be due to under-reporting among heavy drinkers, as well as under-reporting in population groups that are not socially expected to drink in Korea, such as women. Such misclassification could have biased these studies’ results towards lower risk estimates. Conversely, the case–control studies could be prone to recall bias, which could have led to an over-estimation of risks. Although the case–control study on breast cancer [14] reported a statistically significant odds ratio of 1.15 for alcohol drinkers compared to lifetime non-drinkers, which is compatible with the international literature [9], more studies would be needed to generate reliable risk estimates.

## Conclusions

In contrast with several countries in Western Europe, where both alcohol consumption and alcohol-related mortality is decreasing, Korea has the highest per capita alcohol consumption among Asian countries according to a recent report by the World Health Organization [6], and the proportion of lifetime non-drinkers has decreased over the last decade (12.0% in men and 38.9% in women in 1995 vs. 5.1% in men and 20.4% in women between 2001 and 2005) [58]. Excessive alcohol consumption and resulting adverse effects may be attributed to Korean culture, where alcohol drinking is pervasive. Peer persuasion and pressure is very common in Korea when it comes to alcohol consumption, which is considered almost essential in among Korean business people [59]. Furthermore, minors in Korea have relatively easy access to alcohol, hence regulations and control over alcohol consumption need to be strengthened.

Thus, public health initiatives to reduce alcohol consumption, in particular among Korean men, would have a significant impact on cancer incidence and mortality in the Republic of Korea. Among women, awareness of the increased risk of breast cancer due to alcohol consumption may impact drinking behavior.

## Abbreviations

ALDH: Aldehyde dehydrogenases; CI: Confidence interval; PAF: Population attributable fraction; RR: Relative risk.

## Competing interests

The author’s declare that they have no competing interests.

## Authors’ contributions

HRS, MB, PB have made substantial contributions to conception and design; SP, SHJ, SIC, SKP, AS, KWJ, DHL, BL and HRS have contributed to implement the project and acquisition of data and/or analysis of data as well as interpretation of data; SP, AS, KWJ, EW, HRS have been involved in drafting the manuscript or revising it critically for important intellectual content; SP and HRS have given final approval of the version to be published. All authors read and approved the final manuscript.

## Authors’ information

SP worked at the National Cancer Center until February 2012, and is now with the Graduate School of Public Health, at Yonsei University. AS worked at the National Cancer Center until August 2013, and is now with the Seoul National University College of Medicine.

## Pre-publication history

The pre-publication history for this paper can be accessed here:

http://www.biomedcentral.com/1471-2407/14/420/prepub

## Supplementary Material

Additional file 1: Table S1 Prevalence (%) of alcohol drinking in Korea. **Table S2.** Daily alcohol consumption (g/day) in Korea. **Table S3.** Studies included in the meta-analysis for estimating pooled RRs for alcohol drinking on cancer. Click here for file
