# Supplementary material for: Attributable fraction of alcohol consumption on cancer using population-based nationwide cancer incidence and mortality data in the Republic of Korea
Source: BMC Cancer. 2014 Jun 10;14:420. doi: 10.1186/1471-2407-14-420 (PMC4065076; doi:10.1186/1471-2407-14-420)
Supplement: Additional file 1: Table S1 — Prevalence (%) of alcohol drinking in Korea. Table S2. Daily alcohol consumption (g/day) in Korea. Table S3. Studies included in the meta-analysis for estimating pooled RRs for alcohol drinking on cancer. [file 1471-2407-14-420-S1.pdf]

Supplementary Table 1. Prevalence (%) of alcohol drinking in Korea.

**Year**	**Men**	**Women**	**Total**
1989*	77.5	23.5	49.3
1992*	74.6	22.6	46.9
1998**	83.4	54.9	68.4
2001**	82.7	59.5	69.8
2005**	87.2	69.8	78.5

Notes: *Korea National Health Examination Survey. **Korea National Health and Nutrition Examination Survey.

Supplementary Table 2. Daily alcohol consumption (g/day) in Korea.

Average daily alcohol consumption		
Year	Men	Women
**1998**	**28.53**	**6.38**
2001	26.68	5.70
2005	25.33	7.92

Notes: Korea National Health and Nutrition Examination Survey.

Supplementary Table 3. Studies included in the meta-analysis for estimating pooled RRs for alcohol drinking on cancer

Author (year)	Study period	Study subjects					Category of Alcohol intake	OR (95% CI)	Confounding variables considered
		Type and source	Definition	No. of cases	No. of controls	Age mean ±SD			
**Oral cavity**									
Jee SH et al<br>(2010)<br>Re-analysis	1993-2001	Korea Medical<br>Insurance<br>Corporation<br>Age:	Cohort study<br>846,907 men<br>482,618 women	167<br>439<br>92<br>59<br>22		Men:<br>45.2 ± 11.1<br>Women:<br>49.5 ±12.1	0<br>1~24.9<br>25~49.9<br>50~99.9<br>≥100	1.00<br>1.02 (0.85-1.22)<br>1.02 (0.79-1.32)<br>1.27 (0.95-1.72)<br>1.41 (0.91-2.20)	Incidence<br>Adjusted for age, age^2,<br>smoking and diabetes
Jee SH et al<br>(2010)<br>Re-analysis	1993-2001	Korea Medical<br>Insurance<br>Corporation	Cohort study<br>846,907 men<br>482,618 women	31<br>98<br>12<br>11<br>9		Men:<br>45.2 ± 11.1<br>Women:<br>49.5 ±12.1	0<br>1~24.9<br>25~49.9<br>50~99.9<br>≥100	1.00<br>1.58(1.05-2.37)<br>1.33(0.67-2.65)<br>2.49(1.22-5.07)<br>5.84(2.72-12.5)	Mortality<br>Adjusted for age, age^2,<br>smoking and diabetes
Choi SY et al<br>(1991)	1986-1989	Korea Cancer<br>Center Hospital<br>Mean age:	Case/ Control<br>Study<br>Mean age:<br>Case: 55.1(men),<br>51.3(women)	16<br>9<br>45<br>32<br>11	100<br>96<br>90<br>46<br>7	Men:<br>Case:55.1<br>Control:55.4<br>Women<br>Case:51.3<br>Control:51.1	Non-drinker<br>Light<br>Moderate<br>Medium-heavy<br>Heavy	1.00<br>0.59(0.25-1.40)<br>3.61(1.82-7.17)<br>4.23(2.13-8.40)<br>14.82(5.03-43.67)	Incidence<br>Adjusted for cigarette smoking
**Pharynx**									
Choi SY et al<br>(1991)	1986-1989	Korea Cancer<br>Center Hospital	Case/ Control<br>study	16<br>20<br>44<br>40<br>13	100<br>101<br>127<br>63<br>8	Men:<br>Case:53.4<br>Control:53.5<br>Women<br>Case:49.8<br>Control:49.7	Non-drinker<br>Light<br>Moderate<br>Medium-heavy<br>Heavy	1.00<br>1.22(0.60-2.50)<br>2.16(1.13-4.15)<br>4.07(2.11-7.85)<br>11.23(4.23-29.83)	Incidence<br>Adjusted for cigarette smoking
**Esophagus**									
Jee SH et al<br>(2010)<br>Re-analysis	1993-2001	Korea Medical<br>Insurance<br>Corporation	Cohort study<br>846,907 men<br>482,618 women	196<br>854<br>179<br>123<br>42		Men:<br>45.2 ± 11.1<br>Women:<br>49.5 ±12.1	0<br>1~24.9<br>25~49.9<br>50~99.9<br>≥100	1.00<br>1.58(1.05-2.37<br>1.33(0.67-2.65)<br>2.49(1.22-5.07)<br>5.84(2.72-12.50)	Incidence<br>Adjusted for age, age^2,<br>smoking and diabetes
Jee SH et al<br>(2010)<br>Re-analysis	1993-2001	Korea Medical<br>Insurance<br>Corporation	Cohort study<br>846,907 men<br>482,618 women	128<br>548<br>95<br>66<br>23		Men:<br>45.2 ± 11.1<br>Women:<br>49.5 ±12.1	0<br>1~24.9<br>25~49.9<br>50~99.9<br>≥100	1.00<br>2.22(1.82-2.70)<br>3.00(2.28-3.95)<br>4.34(3.19-5.91)<br>4.33(2.76-6.80)	Mortality<br>Adjusted for age, age^2,<br>smoking and diabetes

Supplementary Table 3. Studies included in the meta-analysis for estimating pooled RRs for alcohol drinking on cancer (continued)

Author (year)	Study period	Study subjects					Category of Alcohol intake	OR (95% CI)	Confounding variables considered
		Type and source	Definition	No. of cases	No. of controls	Age mean ±SD			
**Esophagus**									
Kimm H et al (2010)	1992-1995	Korea Medical Insurance Corporation	Cohort study<br>782,632 men	185<br>858<br>184<br>112<br>44		49.0±12.3<br>45.1±11.1<br>42.4±8.6<br>42.0±8.3<br>42.3±8.2	0<br>1~24.9<br>25~49.9<br>50~99.9<br>≥100	1.00<br>2.2(1.9-2.6)<br>3.1(2.5-3.8)<br>3.8(3.0-4.8)<br>4.1(2.9-5.8)	Incidence<br>Adjusted for age, age^2, Alcohol intake, aspartate aminotransferase (GOT), Body mass index, exercise
Kimm H et al (2010)	1992-1995	Korea Medical Insurance Corporation	Cohort study<br>782,632 men	163<br>622<br>110<br>76<br>25		49.0±12.3<br>45.1±11.1<br>42.4±8.6<br>42.0±8.3<br>42.3±8.2	0<br>1~24.9<br>25~49.9<br>50~99.9<br>≥100	1.00<br>1.9(1.6-2.3)<br>2.7(2.1-3.5)<br>3.7(2.8-5.0)<br>3.4(2.2-5.3)	Mortality<br>Adjusted for age, age^2, Alcohol intake, aspartate aminotransferase (GOT), Body mass index, exercise
Yi SW et al (2010)	1985-2005	KangWha County Cohort Study	Cohort study<br>2,696 men<br>3,595wo men	3<br>3<br>4<br>9		55 years or older	None<br>Low<br>Moderate<br>High	1.00<br>1.04(0.17-6.32)<br>2.45(0.53-11.29)<br>5.62(1.45-21.77)	Mortality<br>Adjusted for age, history of chronic disease, smoking habit, ginseng intake, pesticide use, body mass index, education status
Jung EJ et al (2012)	1993-2004	Korean Multi-center Cancer Cohort (KMCC)	Cohort study<br>15,683 men	3<br>1<br>1<br>3<br>3		20- (3.4%)<br>30- (10.8%)<br>40- (19.3%)<br>50- (25.6%)<br>60- (28.8%)<br>70+ (12.1%)	0<br>0.01-25g/week<br>2.01-108g/week<br>108.1-346g/week<br>>346.01 g/week	1.00<br>2.75(0.25-30.51)<br>2.77(0.25-30.98)<br>4.98(0.83-30.04)<br>4.65(0.76-28.39)	Mortality<br>Adjusted for age, BMI and smoking habit
**Colon**									
Jee SH et al (2010) Re-analysis	1993-2001	Korea Medical Insurance Corporation	Cohort study<br>846,907 men<br>482,618 women	1.35<br>2635<br>437<br>253<br>84		Men:<br>45.2 ± 11.1<br>Women:<br>49.5 ±12.1	0<br>1~24.9<br>25~49.9<br>50~99.9<br>≥100	1.00<br>0.98(0.91-1.05)<br>0.76(0.68-0.84)<br>0.85(0.74-0.98)<br>0.84(0.68-1.05)	Incidence<br>Adjusted for age, age^2, smoking and diabetes
Jee SH et al (2010) Re-analysis	1993-2001	Korea Medical Insurance Corporation	Cohort study<br>846,907 men<br>482,618 women	199<br>406<br>50<br>25<br>7		Men:<br>45.2 ± 11.1<br>Women:<br>49.5 ±12.1	0<br>1~24.9<br>25~49.9<br>50~99.9<br>≥100	1.00<br>1.19(1.00-1.42)<br>1.17(0.85-1.61)<br>1.21(0.79-1.86)<br>0.98(0.46-2.10)	Mortality<br>Adjusted for age, age^2, smoking and diabetes
Lim HJ et al. (2008)	1993-1998	Korea Medical Insurance Corporation	Cohort study<br>4,834 men<br>9,470 women	74<br>14<br>8<br>2<br>8		65 years or older	never<br>0< ≤24<br>24< ≤48<br>48< ≤72<br>72<	1.00<br>0.62 (0.35-1.12)<br>1.13 (0.52-2.45)<br>1.07 (0.26-4.48)<br>1.15 (0.28-4.72	Incidence<br>Adjusted for age and sex

Supplementary Table 3. Studies included in the meta-analysis for estimating pooled RRs for alcohol drinking on cancer (continued)

Author (year)	Study period	Study subjects					Category of Alcohol intake	OR (95% CI)	Confounding variables considered
		Type and source	Definition	No. of cases	No. of controls	Age mean ±SD			
**Colon**									
Kim J et al (2009)		Two university hospitals	Case/ Control study	101<br>97<br>163	96<br>82<br>92	30-79 years	<5<br>5-29<br>≥30	1.00<br>1.12(0.73-1.72)<br>1.68(1.13-2.50)	Incidence, Men<br>Crude OR
				217<br>16<br>2	228<br>9<br>2	30-79 years	<5<br>5-29<br>≥-2	1.00<br>1.87(0.76-4.90)<br>1.05(0.08-14.61)	Incidence, Women<br>Crude-OR
Yi SW et al (2010)	1985-2005	KangWha County Cohort Study	Cohort study<br>2,696 men<br>3,595wo men	6<br>8<br>4<br>8		55 years or older	None<br>Low<br>Moderate<br>High	1.00<br>1.57(0.50-4.91)<br>1.33(0.37-4.82)<br>2.61(0.88-7.78)	Mortality<br>Adjusted for age, history of chronic disease, smoking habit, ginseng intake, pesticide use, body mass index, education status
**Liver**									
Jee SH et al (2010)<br>Re-analysis	1993-2001	Korea Medical Insurance Corporation	Cohort study<br>846,907 men<br>482,618 women	1326<br>4608<br>907<br>485<br>215		Men:<br>45.2 ± 11.1<br>Women:<br>49.5 ±12.1	0<br>1~24.9<br>25~49.9<br>50~99.9<br>≥100	1.00<br>0.79(0.74-0.84)<br>0.88(0.80-0.96)<br>0.93(0.84-1.04)<br>1.27(1.09-1.48)	Incidence<br>Adjusted for age, age^2, smoking and diabetes
Jee SH et al (2010)<br>Re-analysis	1993-2001	Korea Medical Insurance Corporation	Cohort study<br>846,907 men<br>482,618 women	766<br>1757<br>454<br>242<br>114		Men:<br>45.2 ± 11.1<br>Women:<br>49.5 ±12.1	0<br>1~24.9<br>25~49.9<br>50~99.9<br>≥100	1.00<br>1.03(0.95-1.12)<br>1.12(1.00-1.26)<br>1.17(1.01-1.35)<br>1.63(1.33-1.98)	Morality<br>Adjusted for age, age^2, smoking and diabetes
Shin HR et al (2004)	1990-1993	Hospital-based (Inje University Pusan Paik Hospital)	Cases: newly histologically confirmed cases<br>Controls: patients of the same hospital	79<br>90<br>34<br>18<br>14<br>9	167<br>194<br>45<br>167<br>194<br>45	Case:<br>53.0 ±8.5<br>Controls:<br>53.1 ±8.5	no<br>moderate<br>heavy<br>no<br>moderate<br>heavy	1.00<br>0.9 (0.7-1.4)<br>1.8 (1.0-3.2)<br>1.00<br>0.6 (0.3-1.4)<br>1.8 (0.8-4.4)	Incidence, HCC cases<br>Matched for sex and age (±4yrs)<br>CLG cases<br>Matched for sex and age (±4yrs)
Yi SW et al (2010)	1985-2005	KangWha County Cohort Study	Cohort study<br>2,696 men<br>3,595wo men	13<br>8<br>8<br>8		55 years or older	None<br>Low<br>Moderate<br>High	1.00<br>0.91(0.38-2.22)<br>0.94(0.38-2.28)<br>0.79(0.31-2.01)	Mortality<br>Adjusted for age, history of chronic disease, smoking habit, ginseng intake, pesticide use, body mass index, education status

Supplementary Table 3. Studies included in the meta-analysis for estimating pooled RRs for alcohol drinking on cancer (continued)

Author (year)	Study period	Study subjects					Category of Alcohol intake	OR (95% CI)	Incidence/Mortality Confounding variables considered
		Type and source	Definition	No. of cases	No. of controls	Age mean ±SD			
**Larynx**									
Jee SH et al (2010) Re-analysis	1993-2001	Korea Medical Insurance Corporation	Cohort study 846,907 men 482,618 women	176<br>530<br>116<br>67<br>23		Men: 45.2 ± 11.1 Women: 49.5 ±12.1	0<br>1~24.9<br>25~49.9<br>50~99.9<br>≥100	1.00<br>1.17(0.98-1.38)<br>1.21(0.96-1.53)<br>1.37(1.03-1.81)<br>1.39(0.90-2.15)	Incidence<br>Adjusted for age, age^2, smoking and diabetes
Jee SH et al (2010) Re-analysis	1993-2001	Korea Medical Insurance Corporation	Cohort study 846,907 men 482,618 women	69<br>180<br>25<br>16<br>8		Men: 45.2 ± 11.1 Women: 49.5 ±12.1	0<br>1~24.9<br>25~49.9<br>50~99.9<br>≥100	1.00<br>1.34(1.01-1.77)<br>1.54(0.96-2.48)<br>2.06(1.18-3.62)<br>2.96(1.40-6.22)	Mortality<br>Adjusted for age, age^2, smoking and diabetes
Choi SY et al (1991)	1986-1989	Korea Cancer Center Hospital	Case/ Control study	16<br>20<br>44<br>40<br>13	100<br>101<br>127<br>63<br>8	Men: Case:58.3 Control:58.8 Women Case:56.0 Control:56.8	Non-drinker<br>Light<br>Moderate<br>Medium-heavy<br>Heavy	1.00<br>1.22(0.60-2.50)<br>2.16(1.13-4.15)<br>4.07(2.11-7.85)<br>11.23(4.23-29.83)	Incidence<br>Adjusted for age, cigarette smoking
**Breast**									
Do MH et al. (2003)	1998-1999	Hospital -based (Hanyang and Soonchunhyang University Hospitals)	Cases: newly histologically confirmed cases Controls: patients of the same hospital	14<br>21<br>14<br>11	8<br>19<br>20<br>20	20-69 years	none<br><45<br>45-149<br>≥150	1.00<br>1.30 (0.72-2.66)<br>0.97 (0.64-3.10)<br>1.49 (0.92-3.28)	Incidence, Premenopausal<br>Adjusted for age, age at menarche, family history of breast cancer, pregnancy, experience of breast feeding, total periods of breastfeeding, total energy intake, fiber intake, vitamin A, C, E intake, and vitamin supplement use
				10<br>15<br>11<br>12	14<br>19<br>12<br>9	20-69 years	none<br><45<br>45-149<br>≥150	1.00<br>1.11(0.34-3.64)<br>1.28(0.35-4.73)<br>1.87(0.49-7.20)	Incidence, Postmenopausal<br>Crude OR

Supplementary Table 3. Studies included in the meta-analysis for estimating pooled RRs for alcohol drinking on cancer (continued)

Author (year)	Study period	Study subjects					Category of Alcohol intake	OR (95% CI)	Confounding variables considered
		Type and source	Definition	No. of cases	No. of controls	Age mean ±SD			
**Breast**									
Kim H et al (2012) Re-analysis		Seoul Breast Cancer Study (SeBCS)	Cases: newly diagnosed histologically breast cancer cases Controls: breast cancer-free population who lived in the community	4,508	4,693		Beta Se(beta)	0.00825* (0.00872) Log(RR) per 10 gram/day alcohol consumption	Incidence Adjusted for age, education, family history, age at menarche, menopausal status, menopausal age, breastfeeding experiences and duration, oral contraceptive use, HRT, BMI, smoking status

* log(OR) per 10g/day alcohol consumption (standard error).

Abbreviations: OR, odds ratio; CI, confidence interval; HBsAg, hepatitis B surface antigen; anti-HCV, antibodies against hepatitis C virus.
